# Cure rate estimation with insufficient follow-up: A median-based bootstrap correction approach

**DOI:** 10.1371/journal.pone.0344669

**Published:** 2026-03-12

**Authors:** Yumiko Ibi, Takashi Omori

**Affiliations:** 1 Department of Biomedical Statistics and Bioinformatics, Kyoto University Graduate School of Medicine, Kyoto, Japan; 2 Department of Clinical Biostatistics, Kyoto University Graduate School of Medicine, Kyoto, Japan; The First Hospital of Jilin University, CHINA

## Abstract

The cure rate in clinical trials can be estimated using the Kaplan–Meier (KM) estimator. However, when the clinical trial follow-up period is insufficient and short, the KM estimator may overestimate the proportion of cured patients. Although a correction method was proposed by Escobar-Bach and Keilegom based on bootstrap sampling, this can also lead to bias when the bootstrap distribution is skewed. We propose a median-based approach for bootstrap samples to address these issues. Simulation results showed that the effect of the variation of the proposed method due to many outliers was smaller than that of the other method and enabled stable estimation. The method was successfully applied to real clinical trial data from a D-penicillamine study on primary biliary cirrhosis.

## 1. Introduction

A cure for an illness is defined as improvement from the illness or injury. In clinical trials with overall survival or progression-free survival as endpoints and in which the follow-up period is limited, participants who do not experience an event of interest during the follow-up period are often regarded as cured patients [1]. They are censored at the end of the trial, and the Kaplan–Meier survival curve shows a long and stable plateau with heavy censoring at the tail [2].

Cure models are used to estimate the cure rate, which is the proportion of the cure rate. There are two types of cure models: mixture and non-mixture cure models [3]. The mixture cure model assumes that trial participants are a mixture of cured and uncured patients. The mixture cure model used in this study is the standard model first introduced by Boag (1949) [4] and later modified by Berkson and Gage (1952) [5] because of its directness and simplicity in interpretation [3]. Mixture cure models also include non-parametric [6,7], semiparametric [8,9], and parametric [10,11] estimations. In semiparametric models, previous studies have proposed transformation models for survival data with cured patients, accounting for measurement error [12,13].

When nonparametrically estimating the cure rate, it is possible to use the estimate from the Kaplan–Meier estimator at the largest observed time. This is possible without bias if the follow-up period is long enough, i.e., if the censoring time is longer than the survival time of uncured patients [14]. In practice, however, the follow-up period of clinical trials may be short or not always sufficiently long until the event of interest occurs [14], and in such situations, the cure rate may be overestimated [15]. To address the issue of overestimating the cure rate in the KM estimator when the follow-up period is insufficient and short, a method adding a correction term to the KM estimator was proposed by Escobar-Bach and Keilegom (2019) [14]. Their method adds a correction term using bootstrap sample means to the KM estimator to estimate the cure rate.

Although the results of their simulation study showed that the bias of their method was reduced compared to that of the KM estimator, the approach can be affected in case of the skewed bootstrap distribution, which may still lead to bias and unstable estimates.

The current study proposes an alternative correction method for estimating cure rates in clinical trials with insufficient or short follow-up periods. Section 2 presents the proposed method and other cure rate estimation methods, Section 3 examines the behavior of the proposed method through simulations, Section 4 applies the proposed method to primary biliary cirrhosis data, and Section 5 provides a discussion.

## 2. Method

### 2.1 Mixture cure model

Let the survival time of the patients be *T* and the censoring time be *C*. *T* and *C* are independent, and *C* is finite. Under the assumption of random right censoring, the observed time is X=min(T,C). Provided that $\tau_{\text{0}}$ is the right endpoint of the survival function for *T* and $\tau_{c}$ is it for C. Here, P[X≤t] denotes the cumulative distribution H(t) of *X*, and $\tau_{H}$ is defined as inf {t: H(t)=1}, the right endpoint of the support of H. $\tau_{H}\;$is the right endpoint of P[X≤t].

A parametric mixture cure model was used in this study. In the model, the survival curve, S(t), is expressed as the cure rate, *p*, and the survival curve of uncured patients, $S_{\text{0}}(t)$, as follows: 0 < *p* < 1.


$$\begin{array}{c} S(t)=p+(\text{1}-p)\;S_{\text{0}}(t) \end{array}$$ (1)

### 2.2 Cure rate estimation methods

The KM estimator may be used to estimate *p* in Equation (1).

The survival times are ordered as follows, $T_{i\;\text{1}\leq i\leq n}$ as $T_{n}^{\left(\text{1}\right)}<T_{n}^{\left(\text{2}\right)}<\cdots <T_{n}^{\left(n\right)}$, where *n* is the sample size. Let $t_{\left(n\right)}$= $T_{n}^{\left(n\right)}$ be the largest observed time among the clinical study participants. One method for estimating the cure rate *p* is to estimate the KM estimator at the largest observed time, which is the KM estimator at the largest observed time.


$$\begin{array}{c} {\hat{p}}_{n}={\hat{S}}_{n}\left(t_{\left(n\right)}\right), \end{array}$$ (2)

where ${\hat{S}}_{n}$ denote the KM estimator of the survival function S(t) based on a sample size n. Escobar-Bach and Keilegom (2019) [14] proposed a method that corrects for the KM estimator as the cure rate calculated by the estimator in Equation (2) may overestimate when the follow-up period is insufficient and short. It is an estimate of *S* ($\tau_{C}$), but the true cure rate, *p* = *S* ($\tau_{\text{0}}$), so this can lead to bias. Their method uses the idea of extreme value theory for the correction term using bootstrap samples. It assumes a survival curve after the censoring time; i.e., it considers what the survival curve would have looked like if followed up after its time. They regarded the largest observed time in a sample as a random variable following an extreme value distribution with parameter *y*, leading to the following formula:


$$\begin{array}{c} {\hat{p}}_{y}={\hat{p}}_{n}-\frac{{\hat{S}}_{n}\left(yt_{\left(n\right)}\right)-{\hat{S}}_{n}\left(t_{\left(n\right)}\right)}{{\hat{y}}_{\gamma }-\text{1}},\;{\hat{y}}_{\gamma }:=\;\frac{{\hat{S}}_{n}\left(yt_{\left(n\right)}\right)-{\hat{S}}_{n}\left(y^{\text{2}}t_{\left(n\right)}\right)}{{\hat{S}}_{n}\left(t_{\left(n\right)}\right)-{\hat{S}}_{n}\left(yt_{\left(n\right)}\right)} \end{array}$$ (3)

where *y* ∈ (0,1) is a tuning parameter.

In Equation (3), −{${\hat{S}}_{n}\left(yt_{\left(n\right)}\right)-{\hat{S}}_{n}\left(t_{\left(n\right)}\right\}/\{{\hat{y}}_{\gamma }-\text{1}\}$ is the correction term. By correcting, $\tau_{C}$ is estimated at $t_{\left(n\right)}$ as S($\tau_{C}$) is close to S($\tau_{\text{0}}$) when *n*→∞. ${\hat{p}}_{y}$ cannot be calculated until *y* is determined. As *y* is not a fixed value and cannot be estimated from the data alone, it must be estimated from the term that best fits the tail function of S(*t*). They used the bootstrap method, estimating a value for *y* of 0.6 to 0.98, and the correction term was calculated as in Equation (4), which is explained in detail in Sections 2.2.2 and 2.2.3.

#### 2.2.1 KM method.

We refer to the estimation method, i.e., the KM estimator at the largest observed time, as the KM method. This was performed without adding a correction term, as estimated in Equation (2).

The following theorem uses the KM method. Assume that 0<p<1 and that S is continuous at $\tau_{H}$ in the case $\tau_{H}<\infty$. Maller and Zhou (1992) states that the estimator ${\hat{p}}_{n}$ is consistent if and only if $\tau_{\text{0}}\leq \tau_{C}$, meaning that, with probability 1, no uncured patients can survive beyond the largest possible censoring time. This condition ensures that sufficient information is available across the entire support of the survival time, so that no observation is almost surely censored. This scenario, commonly referred to as the follow-up period is sufficient, represents the standard paradigm in the analysis of censored data. However, such a condition is not always satisfied in practical studies, particularly when follow-up periods are short or event times are long. When $\tau_{C}<\tau_{\text{0}}$, the follow-up period is insufficient, ${\hat{p}}_{n}$ inevitably underestimates the true cure rate p [14,16].

#### 2.2.2 Escobar and Keilegom method (EK method).

We call the method developed by Escobar-Bach and Keilegom (2019) “EK method”. Since Escobar-Bach and Keilegom (2019) proposed a correction method based on y, we focus on y in this section. In the EK method, *y* is calculated using the mean value of the bootstrap samples in the EK method. Equation (3) is estimated using Equation (4), where $N_{b}$ is the number of bootstrap extractions (200).


$$\begin{array}{c} y^{*}=\text{arg}\underset{y\in H}{\text{min}}|{\hat{p}}_{y}-g\left\{{\hat{p}}_{y^{\left(j\right)}}^{\left(j\right)}\right\}|,\text{ with }y^{\left(j\right)}=\text{sup}\left\{y\in H:{\hat{p}}_{y}^{\left(j\right)}<{\hat{p}}_{n}^{\left(j\right)}\right\},\\ \;j=\text{1},\ldots ,N_{b},\;g\left\{{\hat{p}}_{y^{\left(j\right)}}^{\left(j\right)}\right\}=\frac{\text{1}}{N_{b}}\sum_{j=\text{1}}^{N_{b}}{\hat{p}}_{y^{\left(j\right)}}^{\left(j\right)}, \end{array}$$ (4)

where the choice of *H* = {0.6,0.62,…,0.98} follows Escobar-Bach and Keilegom (2019).

#### 2.2.3 Proposed method (EC method).

The proposed method is a correction to the EK method, called the EK’s correction method “EC method”, which differs from the EK method in terms of the estimation method for *y*. Instead of using the mean of the bootstrap samples, this proposed method uses the median, modifying Equation (4) so that $g\left\{{\hat{p}}_{y^{\left(j\right)}}^{\left(j\right)}\right\}$ changes from $\frac{\text{1}}{N_{b}}\sum_{j=\text{1}}^{N_{b}}{\hat{p}}_{y^{\left(j\right)}}^{\left(j\right)}$ to $\frac{\text{1}}{\text{2}}\left\{{\hat{p}}_{y^{\left(j\right)}}^{\left(j\right)}\left(\frac{N_{b}}{\text{2}}\right)+{\hat{p}}_{y^{\left(j\right)}}^{\left(j\right)}\left(\frac{N_{b}}{\text{2}}+\text{1}\right)\right\}$. This is because the bootstrap samples are a proportion with a value between 0 and 1; therefore, we considered the possibility that the distribution of bootstrap samples could be skewed and affected by variations in the cure rate estimate. Using the median, rather than the mean, may be a more appropriate choice for *y* because of the reduction in the effects of variation and outliers.

### 2.3 Ethical statement

This study used publicly available and fully anonymized data. No new data were collected by the authors. Therefore, ethical approval and informed consent were not required.

## 3. Simulation

### 3.1 Aim and settings

The performance of the proposed cure rate estimation method was evaluated. The purpose was to compare the proposed method with the KM and EK methods through simulations.

The mixture cure model in Equation (1) was used to estimate the cure rate and generate the survival time. The cure rate *p* was assumed to be constant, and four values were considered: 0.2, 0.3, 0.4, and 0.5. As the distribution of $\;S_{\text{0}}(t)$, we used the exponential distribution: exp (−λt). In the survival curves S(t), we considered the following three situations for the follow–up period: (A) insufficient and short, when the plateau of the survival curve was short; (B) insufficient and too short, when the plateau of the survival curve was shorter than (A); and (C) sufficiently long, when the plateau of the survival curve was sufficiently long.

**Fig 1:**
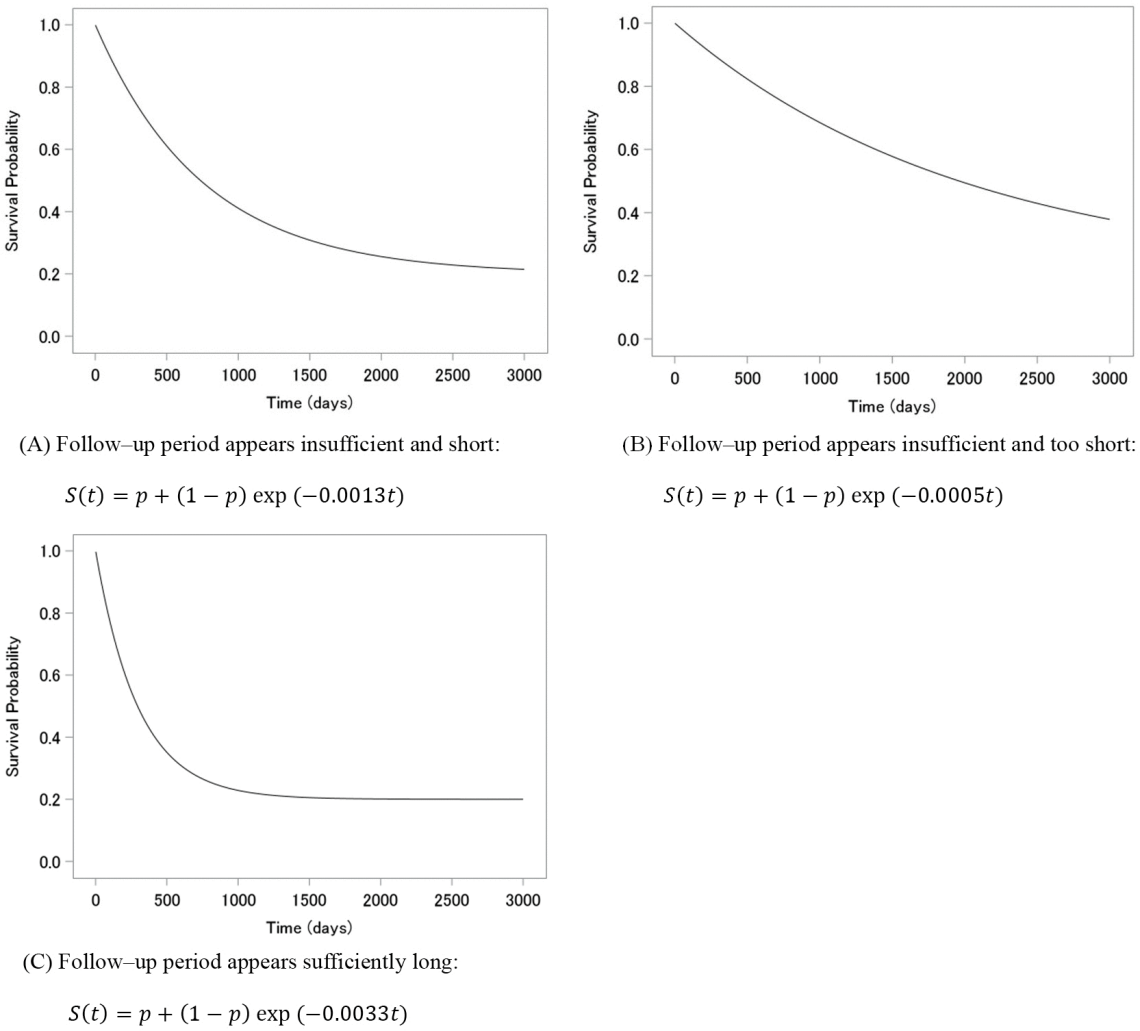
Three Kaplan–Meier curves with different shapes for the true cure rate, 0.2.

The follow–up period was assumed to be 3000 days, and those who survived beyond 3000 days were censored. Fig 1 shows S(t), where the true cure rate is 0.2. In (A), the parameter of the exponential distribution is λ= 0.0013 and the upper 1.83 percentile of the distribution at 3000 days; in (B), λ= 0.0005 and the upper 22.31 percentile; and in (C), λ= 0.0033 and the upper 0.005 percentile.

When implementing the EK and the proposed method, if the correction term could not be calculated, 0 was substituted; if the correction term was less than 0, 0 was substituted; if the correction term exceeded 1, 1 was substituted; and if the estimated cure rate was less than 0, it was 0. The number of simulation repetitions was 1000. The number of bootstrap samples was 200 extraction times (= $N_{b}$) that satisfied $y^{\left(j\right)}=\text{sup}\left\{y\in H:{\hat{p}}_{y}^{\left(j\right)}<{\hat{p}}_{n}^{\left(j\right)}\right\}$ in Equation (4).

Bias, standard deviation (SD), and mean squared error (MSE) were used as indicators to evaluate the simulation. Bias represents the average estimation error, SD represents the variability of the estimates, and MSE represents the mean squared error with respect to the true value. Each indicator was calculated as:


$$\text{Bias}=\frac{\text{1}}{\text{1}\text{0}\text{0}\text{0}}\sum_{i=\text{1}}^{\text{1}\text{0}\text{0}\text{0}}{\hat{p}}_{y*,i}-p,$$

$$\text{SD}=\sqrt{\frac{\text{1}}{\text{1}\text{0}\text{0}\text{0}-\text{1}}\sum_{i=\text{1}}^{\text{1}\text{0}\text{0}\text{0}}{\left({\hat{p}}_{y*,i}-\overset{―}{{\hat{p}}_{y*}}\right)}^{\text{2}}},\;\overset{―}{{\hat{p}}_{y*}}:=\frac{\text{1}}{\text{1}\text{0}\text{0}\text{0}}\sum_{i=\text{1}}^{\text{1}\text{0}\text{0}\text{0}}{\hat{p}}_{y*i},$$

$$\text{MSE}=\frac{\text{1}}{\text{1}\text{0}\text{0}\text{0}}\sum_{i=\text{1}}^{\text{1}\text{0}\text{0}\text{0}}{\left({\hat{p}}_{y*,i}-p\right)}^{\text{2}}.$$

We also examined the distribution of ${\hat{p}}_{y^{\left(j\right)}}^{\left(j\right)},\;$ 200 bootstrap samples in Equations (4) on four typical patterns out of 1000 simulation replications. The four patterns of ${\hat{p}}_{y^{\left(j\right)}}^{\left(j\right)}$, especially at a true cure rate of 0.4, are illustrated in Fig 3. In (a), the difference between the mean and median absolute values of the bootstrap samples was small, and the difference between the EK method and the proposed method was small; in (b), the difference between the absolute values was small, and the difference between the EK method and the proposed method was large; in (c), the difference between the absolute values was large, but the difference between the EK and the proposed methods was small; and in (d), the difference between the absolute values was large and the difference between the EK and the proposed methods was large. The KM estimator for *y* between 0.6 and 0.98 was also considered to examine which of the three survival curves was best for the proposed method.

**Fig 2:**
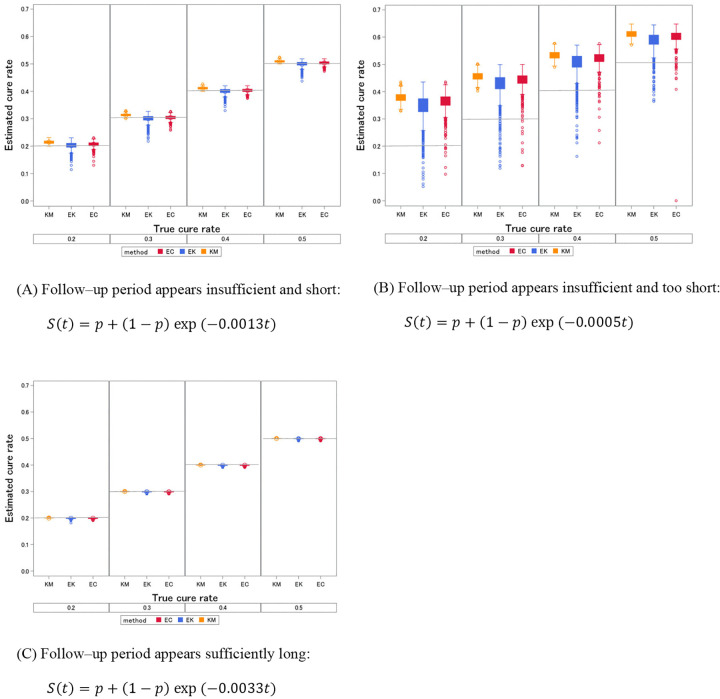
Boxplots of the estimated cure rates for each follow–up period. EC, EK, and KM are the proposed method, and the existing EK and Kaplan–Meier methods, respectively.

All data analyses were performed using SAS version 9.4 (SAS Institute Inc., Cary, NC, USA).

### 3.2 Results

#### 3.2.1 Characteristics of the estimated cure rate.

The box plots in Fig 2 show each setting using the three estimation methods, and the estimated cure rate, Bias, SD, and MSE are summarized in Table 1.

**Table 1:** Estimated cure rate and evaluation indices (Bias, SD, and MSE).

*p*	Estimated cure rate			Bias			SD			MSE		
	KM	EK	EC	KM	EK	EC	KM	EK	EC	KM	EK	EC
(A) Follow–up period appears insufficient and short: $\begin{array}{c} S(t)=p+(1-p)\;exp(-0.0013t) \end{array}$												
0.2	0.215	0.202	0.207	0.0150	0.0020	0.0068	0.0052	0.0126	0.0089	2.5 × 10^−4^	1.6 × 10^−4^	1.2 × 10^−4^
0.3	0.313	0.300	0.304	0.0130	0.0001	0.0042	0.0048	0.0125	0.0079	1.9 × 10^−4^	1.6 × 10^−4^	8.0 × 10^−5^
0.4	0.411	0.400	0.403	0.0112	−0.0000	0.0033	0.0045	0.0104	0.0068	1.5 × 10^−4^	1.1 × 10^−4^	5.7 × 10^−5^
0.5	0.509	0.499	0.503	0.0094	−0.0008	0.0033	0.0042	0.0098	0.0064	1.1 × 10^−4^	9.7 × 10^−5^	5.2 × 10^−5^
(B) Follow–up period appears insufficient and too short: $\begin{array}{c} S(t)=p+(1-p)\;exp(-0.0005t) \end{array}$												
0.2	0.379	0.342	0.362	0.1785	0.1423	0.1620	0.0173	0.0504	0.0327	3.2 × 10^−2^	2.3 × 10^−2^	2.7 × 10^−2^
0.3	0.456	0.423	0.441	0.1564	0.1226	0.1406	0.0158	0.0500	0.0338	2.5 × 10^−2^	1.8 × 10^−2^	2.1 × 10^−2^
0.4	0.534	0.501	0.520	0.1337	0.1006	0.1202	0.0147	0.0481	0.0291	1.8 × 10^−2^	1.2 × 10^−2^	1.5 × 10^−2^
0.5	0.611	0.584	0.599	0.1111	0.0836	0.0993	0.0135	0.0385	0.0297	1.3 × 10^−2^	8.5 × 10^−3^	1.1 × 10^−2^
(C) Follow–up period appears sufficiently long: $\begin{array}{c} S(t)=p+(1-p)\;exp(-0.0033t) \end{array}$												
0.2	0.200	0.199	0.199	0.0001	−0.0011	−0.0009	0.0003	0.0018	0.0015	1.0 × 10^−7^	4.4 × 10^−6^	3.1 × 10^−6^
0.3	0.300	0.299	0.299	0.0000	−0.0010	−0.0010	0.0003	0.0017	0.0018	8.7 × 10^−8^	3.9 × 10^−6^	4.2 × 10^−6^
0.4	0.400	0.399	0.399	0.0000	−0.0009	−0.0009	0.0003	0.0016	0.0015	8.3 × 10^−8^	3.3 × 10^−6^	3.0 × 10^−6^
0.5	0.500	0.499	0.499	0.0000	−0.0008	−0.0007	0.0002	0.0015	0.0013	5.2 × 10^−8^	2.9 × 10^−6^	2.1 × 10^−6^

*p* is the true cure rate, KM is the Kaplan–Meier method, EK is the existing EK method, and EC is the method proposed in this study.

From the box plots in Fig 2, the EK method, which is based on the mean, has much variability. However, the proposed method suppresses the variability of points out of the lower whisker to a greater extent than the EK method. Across all scenarios, the bias was comparable between EK and the proposed method, and SD was the smallest for KM. MSE was the smallest for the proposed method in scenario (A).

#### 3.2.2 Distributions of the four bootstrap samples.

**Fig 3:**
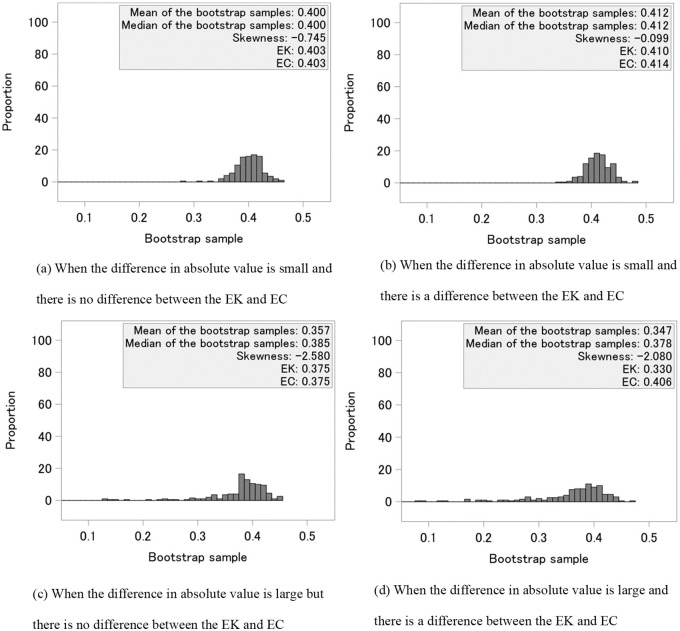
Histograms of bootstrap samples. EK is the existing EK method and EC is the proposed method. The absolute value represents the difference between the mean and the median of the bootstrap samples.

Fig 3 shows histograms of the bootstrap samples. In Fig 3, the mean and median of the bootstrap samples, the skewness of those samples, and the estimated cure rates by EK and EC are shown. Even when the distribution was skewed, there were cases where there was a difference in the estimated cure rate between the EK and the proposed methods, as well as cases where there was no difference.

## 4. Real data application

### 4.1 Aim and settings

The objective was to apply the proposed method to real clinical trial data and examine the behavior of the model. The estimates were calculated using the proposed and EK methods, and the bootstrap sample histogram used for each estimate was examined. The data used were obtained from the D-penicillamine (DPCA) study [17].

This study included a cohort of 312 participants from a placebo-controlled clinical trial of DPCA for primary biliary cirrhosis (PBC) [17,18]. PBC destroys bile ducts in the liver, causing bile accumulation. Progressive tissue damage ultimately leads to liver failure. The time from diagnosis to end-stage liver disease ranges from a few months to 20 years. During the approximate ten-year follow-up period, 125 participants died [17]. Dickson et al. (1989) [18] developed a model to predict survival in patients with PBC using Cox regression analysis and comprehensive data from 312 patients. As previous studies reported no therapeutic differences between control and DPCA treated patients [18], we estimated cure rates using the pooled dataset across the treatment groups.

**Fig 4:**
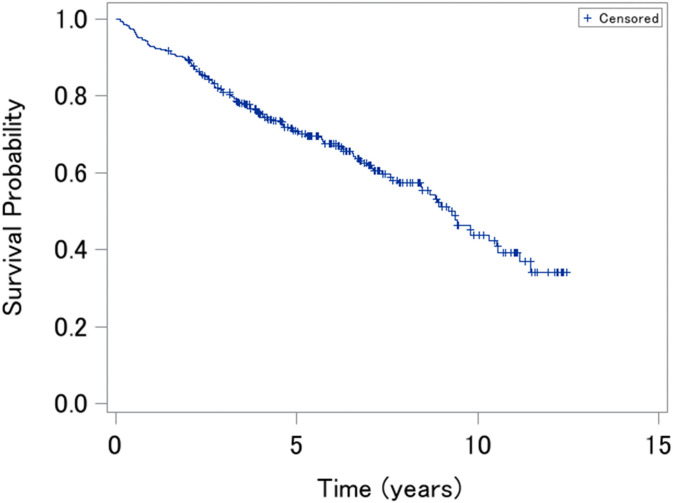
Kaplan–Meier curve for the primary biliary cirrhosis data.

We were interested in the time to death (years) and patients whose status was death as an event occurrence. The Kaplan–Meier curve for the PBC data is presented in Fig 4.

### 4.2 Results

**Fig 5:**
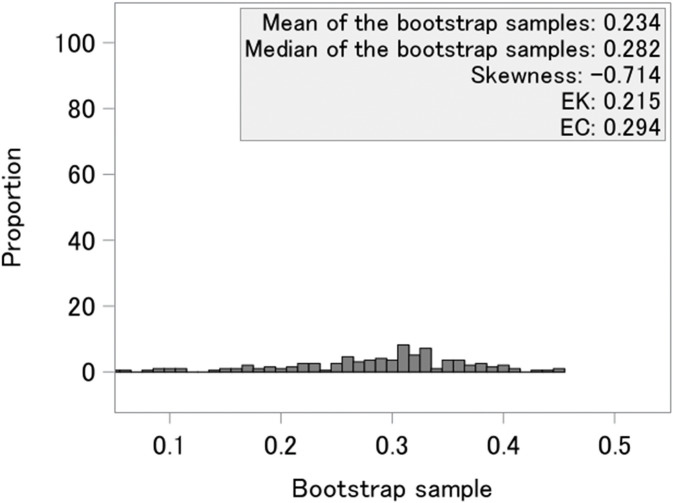
Histogram of bootstrap samples. EK is the existing EK method, and EC is the method proposed in this study.

The results of the estimated cure rate and a histogram of the bootstrap samples of ${\hat{p}}_{y^{\left(j\right)}}^{\left(j\right)}$ are shown in Fig 5. The cure rates estimated by EK was 0.215, the proposed method was 0.294, and that estimated by KM was 0.341. The histogram and skew of −0.714 show mild skewness in the distribution, and there was a difference between the EK and EC.

As suggested by the simulation results, when the bootstrap distribution is skewed, as shown in Fig 5, the cure rate may be overestimated when using the KM estimator, and underestimated when applying the existing mean-based correction method. Although this finding is based on a single data example, it is reasonable to consider the cure rate estimated by the proposed method as a plausible option for evaluating the cure rate. This example further illustrates that, in real applications, estimates of the cure rate can vary depending on the method used.

For practical implementation, it is preferable to examine the histogram of the bootstrap distribution and to assess characteristics such as its mean, median, and skewness. Such evaluations can help determine which estimation method is most appropriate for obtaining the cure rate in each dataset.

## 5. Discussion

In this study, we proposed a cure rate estimation method with a median-based correction term when the clinical trial follow–up period was insufficient and short.

The EC method constructs the correction term based on the median of the bootstrap samples of the estimated survival function. For summarizing the location of asymmetric distributions, the advantages of the median over the mean are established in robust statistics [19]. The median can be interpreted as an M-estimator defined as the minimum value of the following function


$${\hat{\theta }}_{n}=\text{arg }\underset{\theta }{\text{min}}\sum_{i=\text{1}}^{n}|x_{i}-\theta |.$$

Furthermore, when $\{x_{i}{\}}_{i=\text{1}}^{n}$ is regarded as a random sample from a distribution F(x∣θ), the breakdown point—defined as the maximum proportion of contamination that an estimator can tolerate—equals 1/2 for the median, in contrast to 0 for the mean. This provides a theoretical justification for adopting the median rather than the mean when the bootstrap distribution used for bias correction is skewed, a situation that may occur when the bootstrap samples are constructed from estimated survival functions. Moreover, the bootstrap distribution of the mean is also positively skewed, correctly suggesting that the sampling distribution of the mean is asymmetric [20]. In addition, the median is more appropriate than the mean when the data are not normally distributed [21].

The proposed method was more stable as a cure rate estimator than EK, considering point estimates out of the lower whisker in the boxplots in Fig 2 and the MSE in Table 1. As shown in Table 1 in scenario (A), the bias was similar to that of the EK method; however, both methods showed a smaller bias than the KM method as they added a correction to the KM method.

The SD was larger than that of the KM method because of the variation in the bootstrap samples. Regarding the bootstrap samples used in the proposed method, in Section 3.2.2, there were some cases in which there was no difference between the EK and the proposed method in terms of skewness.

Determining whether the follow–up period in clinical trials is sufficient tends to be subjective and ambiguous [22]. The results of this study showed that the corrections led to large variations after the correction in (B) in Fig 2 and an over-reduction of the estimated cure rates in (C). Although the proposed method was developed under the assumption of (A), the circumstances in which it is appropriate must be carefully considered. Table 2 summarizes the estimate of survival function using the Kaplan–Meier method for several y values from the largest observed time (y = 1.0) between 0.6 and 0.98 from the resulting data in 3.2.1, one out of 1000 simulation repetitions, for the true cure rate, 0.2. In Table 2(A), Kaplan–Meier estimators showed minimal change: a small difference between y = 0.6 and y = 0.9 and a negligible difference between y = 0.9 and y = 0.98. In (B), which is set for a follow–up period that is insufficient and too short, the difference between y = 0.6 and y = 0.9 is larger than (A). In (C), there was not much difference between them. In Table 2, a single repetition indicated differences between y = 0.6 and y = 0.9, and y = 0.9 and y = 0.98. To assess the replicability of these findings, we showed the difference in the values of each y from the results of Table 2 (S1 Table) and calculated the proportion of the 1000 repetitions that applied to those differences (S2 Table).

**Table 2:** KM estimator at *y* from the largest observed time (y = 1.0), comparing three survival curves with different parameters of Fig 1, for the true cure rate, 0.2.

Survival curve of Fig 1	Kaplan–Meier estimator at *y* from the largest observed time (y = 1.0)								
	*y* = 0.6	*y* = 0.7	*y* = 0.8	*y* = 0.9	*y* = 0.92	*y* = 0.94	*y* = 0.96	*y* = 0.98	*y =* 1.0
(A)	0.2688	0.2500	0.2396	0.2188	0.2188	0.2188	0.2188	0.2167	0.2167
(B)	0.5479	0.4938	0.4604	0.4208	0.4083	0.3958	0.3917	0.3896	0.3854
(C)	0.2042	0.2042	0.2021	0.2000	0.2000	0.2000	0.2000	0.2000	0.2000

The *y*, as derived from Equations (4), represents the Kaplan–Meier survival estimate at a point from the largest observed time. Follow–up periods (A) and (B) were deemed insufficient, with (B) exhibiting a severely truncated follow–up. Period (C) provided adequate follow–up. If *y* = 1.0, it was consistent with the KM method.

The limitations of this study are as follows. The proposed approach, similar to existing methods, relies on a bootstrap-based correction for an unknown parameter. However, summarizing the location of the bootstrap distribution using either the mean or the median may still yield unstable estimates when extreme values occur, even when the median is applied (Fig 2). Because the aim of the correction is to estimate the unknown parameter appearing in the adjustment term, it may be possible to obtain a more stable and still robust correction by refining how the time point at y times the largest observed time or the censoring time, is incorporated into the estimation procedure. A detailed investigation of such points is beyond the scope of this study and should be addressed in future work. Furthermore, the subjective classification of follow–up periods underscores the need for more objective criteria in future research. Finally, investigations into optimizing the bootstrap resampling process and developing objective criteria for follow–up adequacy would further strengthen the method’s applicability. Exploring the integration of this method with other survival analysis techniques could also broaden its utility in clinical research across diverse disease types and clinical trial designs. The median-based bootstrap correction can enhance the reliability of clinical trial outcomes, particularly in scenarios where prolonged follow–up is impractical, although future studies should explore the method’s performance. Code is available on S1 File.

## Supporting information

S1 Table Supplemental Table1. (DOCX)

S2 Table Supplemental Table2. (DOCX)

S1 File Code. (PDF)
