# Supplementary material for: Cure rate estimation with insufficient follow-up: A median-based bootstrap correction approach
Source: PLoS One. 2026 Mar 12;21(3):e0344669. doi: 10.1371/journal.pone.0344669 (PMC12981499; doi:10.1371/journal.pone.0344669)
Supplement: S1 Table — (DOCX) [file pone.0344669.s001.docx]

**S1 Table.** Difference in the Kaplan–Meier estimator at *y* = 0.6, 0.9, and 0.98

| Survival curve of Figure 1 | Difference | |
| --- | --- | --- |
|  | **Between *y* = 0.6 and *y* = 0.9** | **Between *y* = 0.9 and *y* = 0.98** |
| (A) | 0.050 | 0.002 |
| (B) | 0.127 | 0.031 |
| (C) | 0.004 | 0.000 |

Here is the difference between the KM estimator for *y* = 0.6 and *y* = 0.9 in Table 2 and the difference between the KM estimator for *y* = 0.9 and *y* = 0.98
