# Supplementary material for: Cure rate estimation with insufficient follow-up: A median-based bootstrap correction approach
Source: PLoS One. 2026 Mar 12;21(3):e0344669. doi: 10.1371/journal.pone.0344669 (PMC12981499; doi:10.1371/journal.pone.0344669)
Supplement: S2 Table — (DOCX) [file pone.0344669.s002.docx]

**S2 Table.** The proportion that applies to the difference in the KM estimator at *y* = 0.6, 0.9, and 0.98

| Survival curve of Figure 1 | Between *y* = 0.6 and *y* = 0.9 | | Between *y* = 0.9 and *y* = 0.98 | |
| --- | --- | --- | --- | --- |
|  | **Difference** | **Proportion (%)** | **Difference** | **Proportion (%)** |
| (A) | $0.05$0 | 67.2 | $0.002$ | 43.2 |
| (B) | $0.127$ | 68.3 | $0.031$ | 92.0 |
| (C) | $0.004$ | 94.5 | 0.000 | 100.0 |

Difference indicates the cases where the difference between the KM estimator at *y* = 0.6 and *y* = 0.9 or *y* = 0.9 and *y* = 0.98 is less than or equal to the threshold values for each respective pattern in S1Table. The proportion refers to the proportion of times, out of 1000 simulation repetitions, that the value was less than or equal to each respective difference.
