# Supplementary material for: Cure rate estimation with insufficient follow-up: A median-based bootstrap correction approach
Source: PLoS One. 2026 Mar 12;21(3):e0344669. doi: 10.1371/journal.pone.0344669 (PMC12981499; doi:10.1371/journal.pone.0344669)
Supplement: S1 File — (PDF) [file pone.0344669.s003.pdf]

SAS Ver9.4

```
***********************************************************************************
***********************************************************************************
3. SIMULATION
***********************************************************************************
***********************************************************************************;

%macro cure(p , followuptime , n, number, cure, time);

%do trial = 1 %to 1000;


*******************
*Data
*******************;
data d3;
  call streaminit(&trial. + 213) ;
  do    i = 1 to &n.;
      time = rand ( "exponential" ) * &time. ;
      time = round ( time , 0.1);
      if time >= &followuptime. then censor = 1;
      else censor = 0;
      if censor = 1 then time = &followuptime.;
    OBS = _n_;
    trial = &trial.;
output;
end;
run;

data d2;
  set d3 ( OBS = &cure.) ;
  cure = 1;
run;

data d1;
  merge d3 d2;
  by i;
  if cure = . then cure = 0;
  if cure = 1 then time = &followuptime.;
  if cure = 1 then censor =1;
  drop OBS;
run;

**********
Analysis
**********;
ods select none;
ods listing close;
ods output ProductLimitEstimates = out1 ;
proc lifetest data=d1;
  by trial;
  time time*censor(1);
run;
ods listing;
ods select all;

data out1;
  set out1;
  keep trial survival time Censor;
run;

  data out_retain;
    set out1;
    retain survival2;
    if survival ^ = . then survival2 = survival;
    drop survival;
    rename survival2 = survival;
  run;

proc sort data = out_retain out = out_retain;
  by trial;
run;

*Obtain pn^ in equation (2): the KM estimator at the largest observed time;
data out_last;
  set out_retain end = EOF;
  if EOF = 1 then flag = 1;
  if flag = 1 then output;
  rename survival= pn;
  rename time = tau;
  run;


*Resample the dataset with replacement 200 times using the bootstrap method;
ods select none;
proc surveyselect data = d1 method = urs
  n = &n.
```

```
  seed = 354
  out = strap1
  rep = 200;
  strata trial;
run ;

*Calculate the KM estimator;
ods select none ;
ods output ProductLimitEstimates = out_strap1 ;
proc lifetest data = strap1 ;
  by trial Replicate ;
  time    time * censor (1) ;
  weight NumberHits;
run ;
ods select all ;

data out_strap1;
  set out_strap1;
  keep trial survival time Censor Replicate;
run;

  data out_strap_retain;
    set out_strap1;
    retain survival3;
    if survival ^ = . then survival3 = survival;
    drop survival;
    label Replicate = j;
  rename Replicate =j;
  rename survival3 = survival;
run;

proc sort data = out_strap_retain out =    out_strap_retain;
  by trial j;
run;

*Obtain pn^(j) in equation (4) and (5): the KM estimator at the largest observed time;
data out_strap_last;
  set out_strap_retain;
  by j;
  if last.j then output;
  rename time = tau;
  rename survival = pn;
  trial = &trial.;
run;

data _null_;
    set out_strap_retain end = eof;
    if eof then call symputx ('OBS' , _N_);
run;

%put OBS = &OBS.;

data all_strap;
  set out_strap_retain;
  do H = 0.6 to 0.98 by 0.02 ;
    output;
  end;
run;

proc sort data = all_strap;
  by H ;
run;

proc sort data = all_strap;
  by trial j;
run;


*Merge the dataset for each H with the bootstrap dataset to obtain y in equation (4) and (5) later;
data merge_strap;
  merge all_strap out_strap_last;
  by trial j ;
run;

*Conduct for the bootstrap data and non-bootstrap (usual) data;
%macro correction (data = , y =    );

*Add time points scaled by yt(n) and y*yt(n) to the dataset, and mark flags for all time points before yt(n) and y*yt(n), respectively
to estimate Sn^(yt(n)) and Sn^(y*yt(n)) in equation (3);
data merge_&data.;
  set merge_&data.;
  ytau = H*tau;
  yytau = H*H*tau;
  if time <= ytau then ytauflag = 1;
  if time <= yytau then yytauflag = 1;
run;

*Get the last observation of ytauflag;
```

```
data yflagmerge_&data.;
  set merge_&data.;
  where ytauflag ^=. ;
run;

*Extract observations with yflag = 1;
data yflagmerge_&data.;
  set yflagmerge_&data.;
  by trial j H ytauflag;
  if last.ytauflag then yflag = 1;
run;

data yflag_&data.;
  set yflagmerge_&data.;
  where yflag = 1;
  rename survival = sytau;      *Sn(ytauj)=jsytau;
run;

*Get the last observation of yytauflag;
data yyflagmerge_&data.;
  set merge_&data.;
  where yytauflag ^=. ;
run;

*Extract observations with yyflag = 1;
data yyflagmerge_&data.;
  set yyflagmerge_&data.;
  by trial j H yytauflag;
  if last.yytauflag then yyflag = 1;
run;

data yyflag_&data.;
  set yyflagmerge_&data.;
  where yyflag = 1;
  rename survival = syytau;
run;

data final_&data.;
  merge yflag_&data. yyflag_&data.;
  by trial j H;
  keep trial j tau H    sytau syytau pn Censor;
run;

*Caluculate {Sn^(yt(n)) - Sn^(t(n))} / (yγ^-1) in equation (3): correction term;
data final3_&data.;
  set final_&data.;
  gamma_up = round( ( sytau - syytau) , 0.00000001); *Sn^(yt(n)) - Sn^(y*yt(n));
  gamma_low = round( (pn    - sytau) ,0.00000001) ; *Sn^(t(n))-Sn^(yt(n));
  gamma = round( ( gamma_up / gamma_low) ,0.00000001) ; *yγ^;
  if gamma = . then gamma = 0 ;
  y_cor_up = round( ( sytau - pn) ,0.00000001) ; *Sn^(yt(n)) - Sn^(t(n));
  y_cor_low = round( ( gamma-1) ,0.00000001) ; *(yγ^-1);
  y_cor = round( ( y_cor_up / y_cor_low) ,0.00000001) ; *{Sn^(yt(n)) - Sn^(t(n))} / (yγ^-1);
  if y_cor = . then y_cor = 0;
  if y_cor < 0 then y_cor = 0;
  if y_cor > 1 then y_cor = 1;
run;

*Caluculate py^;
data final1_&data.;
  set final3_&data.;
  py = pn - y_cor;
  if py < 0 then py = 0;
  if py > 1 then py = 1;
  y_sup = py - pn;
run;

%mend;
%correction (data = strap );

proc sort data = final1_strap out = final1_strap;
  by trial j y_sup;
run;

*Limit to those satisfying condition py^(j) < pn^(j) in equation (5);
data aaa;
  set final1_strap;
  if y_sup >= 0 then delete;
run;

proc sort data = aaa out = bbb;
  by trial j H;
run;

*Obtain py(j)^(j) in equation (4) and (5);
data ccc;
  set bbb;
  by trial j H;
```

```
  if last.j then output;
run;

data ddd;
  set ccc;
  rename py = cor_pyj;
run;

*Calculate g{py(j)^(j)}: the mean and median of the py(j)^(j) in equation (4) and (5);
ods select none;
proc means data = ddd mean median skew kurt;
  class trial;
  var cor_pyj;      *py(j)^(j) in equation (4) and (5);
  output out = sub mean = cor_pyj_mean median = cor_pyj_median skew = skew kurt = kurt;          *cor_pyj_mean and cor_pyj_median: g{py(j)^(j)} in equation (4) and (5);
run;
ods select all;

data sub;
  set sub;
  where trial ^ = . ;
  drop _TYPE_ _FREQ_;
run;

data skewcal;
  set sub;
  minus = cor_pyj_mean - cor_pyj_median;
  pro = cor_pyj_mean/cor_pyj_median;
run;

data _null_;
  set out_retain end = eof;
  if eof then call symputx ('OBS' , _N_);
run;

%put OBS = &OBS.;

data all_regular;
  set out_retain;
  j = .;
  do H = 0.6 to 0.98 by 0.02 ;
    output;
    end;
run;

proc sort data = all_regular;
  by H;
run;

data merge_usual;
  merge all_regular out_last;
  by trial ;
run;

%correction (data = usual);

*Calculate py^ by H;
data ystr;
  merge final1_usual sub;
  by trial;
run;

* Calculate to determine y in equation (3);
data ystr;
  set ystr;
  where tau^ = . ;
  y_right = pn - y_cor;
  y_right = round(y_right , 0.00000001);
  y_str_mean = abs(y_right - cor_pyj_Mean); *|py^ - g{py(j)^(j)}| in equation (4);
  y_str_mean = round( y_str_mean , 0.00000001);
  y_str_median = abs(y_right - cor_pyj_Median); *|py^ - g{py(j)^(j)}| in equation (5);
  y_str_median = round( y_str_median , 0.00000001);
  drop sytau tau syytau;
run;

proc sort data=ystr out=ystr_mean;
  by trial y_str_mean H;
run;

proc sort data=ystr out=ystr_median;
  by trial y_str_median H;
run;

data ystr2_mean;
  set ystr_mean;
  by trial;
  if first.trial then output;
  rename y_str_mean = y_deter_mean;
  rename H = H_mean;
```

```
  rename gamma_up = gamma_up_mean;
  rename gamma_low = gamma_low_mean;
  rename gamma = gamma_mean;
  rename y_cor_up = y_cor_up_mean;
  rename y_cor_low = y_cor_low_mean;
  rename y_cor = y_cor_mean;
run;


data ystr2_median;
  set ystr_median;
  by trial;
  if first.trial then output;
  rename y_str_median = y_deter_median;
  rename H = H_median;
  rename gamma_up = gamma_up_median;
  rename gamma_low = gamma_low_median;
  rename gamma = gamma_median;
  rename y_cor_up = y_cor_up_median;
  rename y_cor_low = y_cor_low_median;
  rename y_cor = y_cor_median;
run;

data ystr3_mean;
  merge ystr_mean ystr2_mean;
  by trial ;
run;

data ystr3_median;
  merge ystr_median ystr2_median;
  by trial ;
run;

data ystr4_mean;
  set ystr3_mean;
  where y_str_mean = y_deter_mean;
  by trial H;
  if last.trial then output;
  rename y_right = y_right_EK;
run;

data ystr4_median;
  set ystr3_median;
  where y_str_median = y_deter_median;
  by trial H;
  if last.trial then output;
  rename y_right = y_right_EK_cor;
run;

data ystr5;
  merge ystr4_mean ystr4_median;
  by trial;
run;

*Equation (3) with y in equation (4) and (5);
*Obtain cure rate estimation: "pn" = KM, "y_right_EK" = EK, "y_right_EK_cor" = EC;
data ystr5;
  format trial Censor    y_sup H_mean H_median
                         cor_pyj_mean cor_pyj_median y_deter_mean y_deter_median
                         gamma_up_mean    gamma_up_median gamma_low_mean    gamma_low_median gamma_mean    gamma_median
                         y_cor_up_mean y_cor_up_median    y_cor_low_mean y_cor_low_median y_cor_mean y_cor_median pn y_right_EK y_right_EK_cor;
  set ystr5;
  if y_right_EK_cor < 0 then y_right_EK_cor = 0;
  else if y_right_EK_cor > 1 then y_right_EK_cor = 1;
  if y_right_EK < 0 then y_right_EK = 0;
  else if y_right_EK > 1 then y_right_EK = 1;
  drop j H py y_str_mean y_str_median gamma_up gamma_low gamma y_cor_up y_cor_low y_cor;
run;

proc append base = append_&number. data = ystr5;
run;

proc append base = append_boot&number. data = ddd;
run;

proc append base = append_skewcal&number. data = skewcal;
run;

%end;

proc export data = append_&number. outfile = "xxx.&time.&p.ystr5.dataset.csv"
  dbms = csv replace;
run;

proc export data = append_boot&number. outfile = "xxx.&time.&p.ddd.dataset.csv "
  dbms = csv replace;
run;
```

```
proc export data = append_skewcal&number. outfile = "xxx.&time.&p.skewcal.dataset.csv"
  dbms = csv replace;
run;


%mend cure;

*time = 750: the follow-up period appears insufficient and short (A);
*Apply the same code with the following time values:
    time = 300 - (B) the follow-up period appears sufficiently long
    time = 2000 - (C) the follow-up period appears sufficiently long;
%cure (p = 0.2 , followuptime = 3000 , n =480, number =3 ,cure = 96, time = 750);
%cure (p = 0.3 , followuptime = 3000 , n =480, number =6 ,cure = 144 , time = 750);
%cure (p = 0.4 , followuptime = 3000 , n =480, number =9 ,cure = 192, time = 750);
%cure (p = 0.5 , followuptime = 3000 , n =480, number =12 ,cure = 240, time = 750);


*********************************
*********************************
Table1
*********************************
*********************************;

****************
Data;
****************;
*Import data for the three follow-up scenarios (Figure 1(A)–(C));
proc import out=estimate3
  datafile="xxx.0.2.ystr5.dataset.csv"
  dbms=csv replace;
  getnames=yes;
  datarow=2;
run;

proc import out=estimate6
  datafile="xxx.0.3.ystr5.dataset.csv"
  dbms=csv replace;
  getnames=yes;
  datarow=2;
run;

proc import out=estimate9
  datafile="xxx.0.4.ystr5.dataset.csv"
  dbms=csv replace;
  getnames=yes;
  datarow=2;
run;

proc import out=estimate12
  datafile="xxx.0.5.ystr5.dataset.csv"
  dbms=csv replace;
  getnames=yes;
  datarow=2;
run;

**********
Analysis
**********;
%macro evaluation (senario = , curerate = );

  data estimate2&senario.;
  set estimate&senario.;
  pnbias =    (pn - &curerate.);
  EKbias = ( y_right_EK- &curerate.);
  EK_corbias =    ( y_right_EK_cor- &curerate.);
  pnrootMSE = (pn - &curerate.)*(pn - &curerate.);
  EKrootMSE =( y_right_EK- &curerate.)*( y_right_EK- &curerate.);
  EKcoerootMSE =( y_right_EK_cor- &curerate.)*(y_right_EK_cor- &curerate.);
run;

proc means data = estimate2&senario. mean;
  var pn y_right_EK y_right_EK_cor;
  output out = mean&senario.;
run;

proc means data = estimate2&senario. mean;
  var pnbias EKbias EK_corbias ;
  output out = sub_mean&senario.;
run;

proc means data = estimate2&senario. std;
  var pn y_right_EK y_right_EK_cor ;
  output out = sub_std&senario.;
run;

proc means data = estimate2&senario. mean;
  var pnrootMSE EKrootMSE EKcoerootMSE ;
  output out = sub_MSE&senario.;
```

```
run;

data mean2&senario.;
  set mean&senario.;
  where _STAT_ = "MEAN";
run;

data sub2_mean&senario.;
  set sub_mean&senario.;
  where _STAT_ = "MEAN";
run;

data sub2_std&senario.;
  set sub_std&senario.;
  where _STAT_ = "STD";
run;

data sub3_std&senario.;
  set sub2_std&senario.;
  rename pn = pnstd;
  rename y_right_EK = y_right_EKstd;
  rename y_right_EK_cor = y_right_EK_corstd;
run;

data sub2_mse&senario.;
  set sub_mse&senario.;
  where _STAT_ = "MEAN";
run;

data evaluation&senario.;
  merge mean2&senario. sub2_mean&senario. sub3_std&senario. sub2_mse&senario.;
  senario = &senario.;
  p = &curerate.;
run;

*"pn" = KM, "y_right_EK" = EK, "y_right_EK_cor" = EC;
proc append base = evalationindex2 data = evaluation&senario.;
run;

%mend evaluation;
%evaluation ( senario = 3 , curerate = 0.2);
%evaluation ( senario = 6 , curerate = 0.3);
%evaluation ( senario = 9 , curerate = 0.4);
%evaluation ( senario = 12 , curerate = 0.5);


*********************************
*********************************
Table2
*********************************
*********************************;

*Summary of sytau: KM estimator at y from the "final_usual" dataset when p = 0.2, generated during the simulation;
*For each of the three follow-up scenarios (Figure 1(A)～(C));



*********************************
*********************************
Figure3
*********************************
*********************************;

****************
*Data
****************;
*import data when the follow-up period appears insufficient and short: Figure1(A);
*p = 0.4;
proc import out=d4
  datafile="xxx.0.4.ddd.dataset.csv"
  dbms=csv replace;
  getnames=yes;
  datarow=2;
run;


*From the 1000 iterations, keep only the four patterns required for Figure 3;
data d4necessary;
  set d4;
  if trial = 221 then output;
  else if trial = 344 then output;
  else if trial = 611 then output;
  else if trial = 502 then output;
run;

data d4necessary221;
```

```
  set d4necessary;
  if trial = 221 then output;
run;
*Create similar datasets for 502, 344, and 611;


**********
Analysis
**********;
*Figure3(a): Generate a histogram for variable cor_pyj in d4necessary221 dataset to visualize its distribution.
*The same analysis is applied to the following trials:
      trial = 502-Figure 3(b)
      trial = 344-Figure 3(c)
      trial = 611-Figure 3(d);




*********************************
*********************************
4. REAL DATA APPLICATION
*********************************
*********************************

****************
Data
****************;
proc import out=pbc
  datafile="xxx.csv"
  dbms=csv replace;
  getnames=yes;
  datarow=2;
run;

data pbc1;
  set pbc;
  if status = "Dead" then censor = 1;
  else if status = "Censored" then censor = 0;
  rename years = time;
run;

**********
Analysis
**********;
*Caluculate the Kaplan-Meier (KM) estimator;
ods select none;
ods listing close;
ods output ProductLimitEstimates = out1 ;
proc lifetest data=pbc1;
  time time*censor(0);
run;
ods listing;
ods select all;

data out1;
  set out1;
  keep survival time Censor;
run;

  data out_retain;
    set out1;
    retain survival2;
    if survival ^ = . then survival2 = survival;
    drop survival;
    rename survival2 = survival;
  run;

*Obtain pn^ in equation (2): the KM estimator at the largest observed time;
data out_last;
  set out_retain end = EOF;
  if EOF = 1 then flag = 1;
  if flag = 1 then output;
  rename survival= pn; *pn^;
  rename time = tau; *t(n);
  run;

*Resample the dataset with replacement 200 times using the bootstrap method;
ods select none;
proc surveyselect data = pbc1 method = urs
  n = 312
  seed = 354
  out = strap1
  rep = 200;
run ;

*Calculate the KM estimator;
ods select none ;
ods output ProductLimitEstimates = out_strap1 ;
```

```
proc lifetest data = strap1 ;
  by Replicate ;
  time    time * censor (0) ;
  weight NumberHits;
run ;
ods select all ;

data out_strap1;
  set out_strap1;
  keep survival time Censor Replicate;
run;

  data out_strap_retain;
    set out_strap1;
    retain survival3;
    if survival ^ = . then survival3 = survival;
    drop survival;
    label Replicate = j;
  rename Replicate =j;
  rename survival3 = survival;
run;

proc sort data = out_strap_retain out =    out_strap_retain;
  by j;
run;

*Obtain pn^(j) in equation (4) and (5): the KM estimator at the largest observed time;
data out_strap_last;
  set out_strap_retain;
  by j;
  if last.j then output;
  rename time = tau;
  rename survival = pn;
run;

  data _null_;
    set out_strap_retain end = eof;
    if eof then call symputx ('OBS' , _N_);
run;

%put OBS = &OBS.;

data all_strap;
  set out_strap_retain;
  do H = 0.6 to 0.98 by 0.02 ;
    output;
  end;
run;

proc sort data = all_strap;
  by H ;
run;

proc sort data = all_strap;
  by j;
run;

*Merge the dataset for each H with the bootstrap dataset to obtain y in equation (4) and (5) later;
data merge_strap;
  merge all_strap out_strap_last;
  by j ;
run;

*Conduct for the bootstrap data and non-bootstrap (usual) data;
%macro correction (data = , y =    );

*Add time points scaled by yt(n) and y*yt(n) to the dataset, and mark flags for all time points before yt(n) and y*yt(n), respectively
to estimate Sn^(yt(n)) and Sn^(y*yt(n)) in equation (3);
data merge_&data.;
  set merge_&data.;
  ytau = H*tau; *yt(n);
  yytau = H*H*tau; *y*yt(n);
  if time <= ytau then ytauflag = 1;
  if time <= yytau then yytauflag = 1;
run;

data yflagmerge_&data.;
  set merge_&data.;
  where ytauflag ^=. ;
run;

*Get the last observation of ytauflag;
data yflagmerge_&data.;
  set yflagmerge_&data.;
  by j H ytauflag;
  if last.ytauflag then yflag = 1;
run;
```

```
*Extract observations with yflag = 1;
data yflag_&data.;
  set yflagmerge_&data.;
  where yflag = 1;
  rename survival = sytau;
run;

data yyflagmerge_&data.;
  set merge_&data.;
  where yytauflag ^=. ;
run;

*Get the last observation of yytauflag;
data yyflagmerge_&data.;
  set yyflagmerge_&data.;
  by    j H yytauflag;
  if last.yytauflag then yyflag = 1;
run;

*Extract observations with yyflag = 1;
data yyflag_&data.;
  set yyflagmerge_&data.;
  where yyflag = 1;
  rename survival = syytau;
run;

data final_&data.;
  merge yflag_&data. yyflag_&data.;
  by j H;
  keep j tau H    sytau syytau pn Censor;
run;

*Caluculate {Sn^(yt(n)) - Sn^(t(n))} / (yγ^-1) in equation (3): correction term;
data final1_&data.;
  set final_&data.;
  gamma_up = round( ( sytau - syytau) , 0.00000001); *Sn^(yt(n)) - Sn^(y*yt(n));
  gamma_low = round( (pn    - sytau) ,0.00000001) ; *Sn^(t(n))-Sn^(yt(n));
  gamma = round( ( gamma_up / gamma_low) ,0.00000001) ; *yγ^;
  if gamma = . then gamma = 0 ;
  y_cor_up = round( ( sytau - pn) ,0.00000001) ; *Sn^(yt(n)) - Sn^(t(n));
  y_cor_low = round( ( gamma-1) ,0.00000001) ; *(yγ^-1);
  y_cor = round( ( y_cor_up / y_cor_low) ,0.00000001) ; *{Sn^(yt(n)) - Sn^(t(n))} / (yγ^-1);
  if y_cor = . then y_cor = 0;
  if y_cor < 0 then y_cor = 0;
  if y_cor > 1 then y_cor = 1;
run;

*Caluculate py^;
data final3_&data.;
  set final1_&data.;
  py = pn - y_cor;      *py^ in equation (3);
  if py < 0 then py = 0;
  if py > 1 then py = 1;
  y_sup = py - pn; *py^(j) - pn^(j);
run;

%mend;
%correction (data = strap );

proc sort data = final3_strap out = final3_strap;
  by j y_sup;
run;

*Limit to those satisfying condition py^(j) < pn^(j) in equation (5);
data final4_strap;
  set final3_strap;
  if y_sup >= 0 then delete;
run;

proc sort data = final4_strap out = final5_strap;
  by j H;
run;

*Obtain py(j)^(j) in equation (4) and (5);
data final6_strap;
  set final5_strap;
  by j H;
  if last.j then output;
run;

data final7_strap;
  set final6_strap;
  rename py = cor_pyj;
run;

*Calculate g{py(j)^(j)}: the mean and median of the py(j)^(j) in equation (4) and (5);
ods select none;
proc means data = final7_strap mean median skew kurt;
```

```
  var cor_pyj;    *py(j)^(j) in equation (4) and (5);
  output out = sub mean = cor_pyj_mean median = cor_pyj_median skew = skew kurt = kurt;    *cor_pyj_mean and cor_pyj_median: g{py(j)^(j)} in equation (4) and (5);
run;
ods select all;

data sub;
  set sub;
  drop _TYPE_ _FREQ_;
run;

  data _null_;
    set out_retain end = eof;
    if eof then call symputx ('OBS' , _N_);
run;

%put OBS = &OBS.;

data all_regular;
  set out_retain;
    j = .;
  do H = 0.6 to 0.98 by 0.02 ;
    output;
    end;
run;

proc sort data = all_regular;
  by H;
run;

data merge_usual;
  set all_regular;
  if _N_ = 1 then set out_last;
run;

%correction (data = usual);

*Calculate py^ by H;
data ystr;
  set final1_usual;
  if _N_ = 1 then set sub;
run;

* Calculate to determine y in equation (3);
data ystr;
  set ystr;
  where tau^ = . ;
  y_right = pn - y_cor; *py^;
  y_right = round(y_right , 0.00000001);
  y_str_mean = abs(y_right - cor_pyj_Mean); *|py^ - g{py(j)^(j)}| in equation (4);
  y_str_mean = round( y_str_mean , 0.00000001);
  y_str_median = abs(y_right - cor_pyj_Median); *|py^ - g{py(j)^(j)}| in equation (5);
  y_str_median = round( y_str_median , 0.00000001);
  drop sytau tau syytau;
run;

proc sort data=ystr out=ystr_mean;
  by y_str_mean H;
run;

proc sort data=ystr out=ystr_median;
  by y_str_median H;
run;

data ystr2_mean;
  set ystr_mean;
  rename y_str_mean = y_deter_mean;
  rename H = H_mean;
  rename gamma_up = gamma_up_mean;
  rename gamma_low = gamma_low_mean;
  rename gamma = gamma_mean;
  rename y_cor_up = y_cor_up_mean;
  rename y_cor_low = y_cor_low_mean;
  rename y_cor = y_cor_mean;
run;

data ystr2_median;
  set ystr_median;
  rename y_str_median = y_deter_median;
  rename H = H_median;
  rename gamma_up = gamma_up_median;
  rename gamma_low = gamma_low_median;
  rename gamma = gamma_median;
  rename y_cor_up = y_cor_up_median;
  rename y_cor_low = y_cor_low_median;
  rename y_cor = y_cor_median;
run;

data ystr3_mean;
```

```
  merge ystr_mean ystr2_mean;
run;

proc sort data =    ystr3_mean;
  by y_str_mean H;
run;

data ystr3_median;
  merge ystr_median ystr2_median;
run;

proc sort data =    ystr3_median;
  by y_str_median H;
run;

data ystr4_mean;
  set ystr3_mean (obs = 1);
  where y_str_mean = y_deter_mean;
  by y_str_median H;
  if first.H then output;
  rename y_right = y_right_EK;
run;

data ystr4_median;
  set ystr3_median (obs = 1);
  where y_str_median = y_deter_median;
  by y_str_mean H;
  if first.H then output;
  rename y_right = y_right_EK_cor;
run;

data ystr5;
  merge ystr4_mean ystr4_median;
run;

*Equation (3) with y in equation (4) and (5);
*Obtain cure rate estimation: "pn" = KM, "y_right_EK" = EK, "y_right_EK_cor" = EC;
data ystr5;
  format Censor    H_mean H_median
                cor_pyj_mean cor_pyj_median y_deter_mean y_deter_median
                gamma_up_mean    gamma_up_median gamma_low_mean    gamma_low_median gamma_mean    gamma_median
                y_cor_up_mean y_cor_up_median    y_cor_low_mean y_cor_low_median y_cor_mean y_cor_median pn y_right_EK y_right_EK_cor;
  set ystr5;
  if y_right_EK_cor < 0 then y_right_EK_cor = 0;
  else if y_right_EK_cor > 1 then y_right_EK_cor = 1;
  if y_right_EK < 0 then y_right_EK = 0;
  else if y_right_EK > 1 then y_right_EK = 1;
  drop j H    y_str_mean y_str_median gamma_up gamma_low gamma y_cor_up y_cor_low y_cor ;
run;
```
